# Single internal carotid cavernous sinus aneurysm presented as bilateral painful ophthalmoplegia: a case report

**DOI:** 10.1186/s12883-023-03066-0

**Published:** 2023-01-17

**Authors:** Wanwan Zhang, Yinyin Xie, Jiwei Zhang, Cui Wang, Yajun Lian, Hongbo Liu, Nanchang Xie

**Affiliations:** 1 Department of Neurology, The First Affliated Hospital of Zhengzhou University, 450052 Zhengzhou, China; 2 Academy of Medical Sciences, Zhengzhou University, Zhengzhou, 450052 China; 3 Department of Clinical Laboratory, Key Clinical Laboratory of Henan Province, The First Affiliated Hospital of Zhengzhou University, Zhengzhou, 450052 China

**Keywords:** Aneurysm, Painful ophthalmoplegia, Cavernous sinus, Internal carotid artery, Aneurysm embolization

## Abstract

**Background:**

Intracranial aneurysms are the most common vascular cause of painful ophthalmoplegia. Symptoms include retro-orbital pain, diplopia, ophthalmoplegia, trigeminal neuropathy, or a combination of these. Most single aneurysms cause ipsilateral, painful ophthalmoplegia. Here, we report the first, to our knowledge, case of bilateral painful ophthalmoplegia possibly caused by an aneurysm of the cavernous segment of the left internal carotid artery.

**Case presentation:**

A 62-year-old male patient presented with headache and bilateral ptosis. Laboratory tests revealed hypopituitary function. Computerized tomography angiography showed a large aneurysm in the cavernous sinus segment of the left internal carotid artery. Aneurysm embolization was performed in the Nerve Interventional Department. Four months after surgery, the patient's symptoms returned to normal.

**Conclusions:**

This case suggests that patients with bilateral painful ophthalmoplegia should be screened for aneurysms using computed tomography angiography or magnetic resonance angiography immediately.

## Background

Painful ophthalmoplegia, which involves periorbital or hemicranial pain with paralysis of ipsilateral extraocular motility, can have various causes, including trauma, neoplasms, vascular and inflammatory diseases [1]. Intracranial aneurysms are the most common vascular cause, which can compress the cranial nerves III-VI and cause retro-orbital pain, diplopia, ophthalmoplegia, trigeminal neuropathy, or a combination of the above [2]. Bilateral painful ophthalmoplegia is rare, and is mostly caused by infectious diseases, ophthalmoplegia migraine, cavernous sinus thrombosis, or intracranial hypotension [3–6]. Herein, we report the first case of bilateral painful ophthalmoplegia possibly caused by an aneurysm of the cavernous segment of the left internal carotid artery.

## Case presentation

**Fig. 1:**
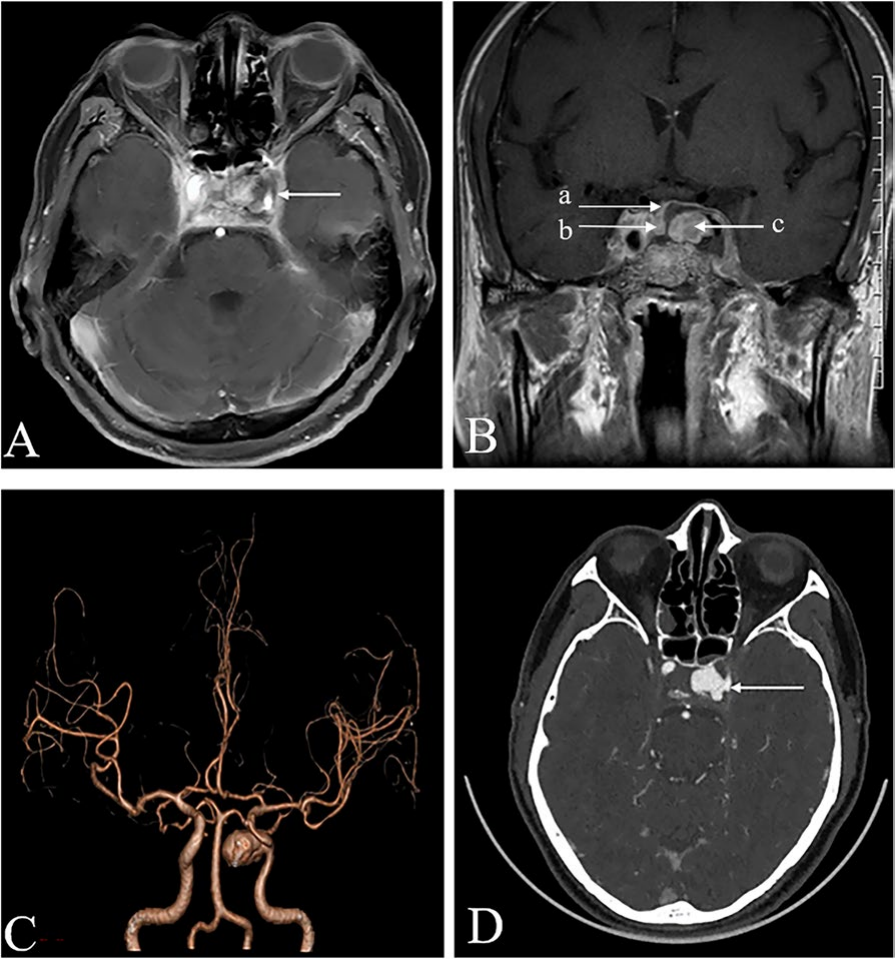
**A**: an axial contrast-enhanced magnetic resonance image reveals a mass lesion in the left cavernous sinus (arrow). **B**: Pituitary contrast-enhanced T1-weighted magnetic resonance image shows pituitary compression by the aneurysm (arrow **c**) and a right shift of pituitary stalk under compression (arrow **a**). Arrow** b** shows the pituitary gland. **C**: Computed tomography angiography shows a large aneurysm in the cavernous sinus segment of the left internal carotid artery about 14.2 × 11.7 mm. **D**: an axial raw image of computed tomography angiography at the level of biggest diameter of the aneurysm

**Fig. 2:**
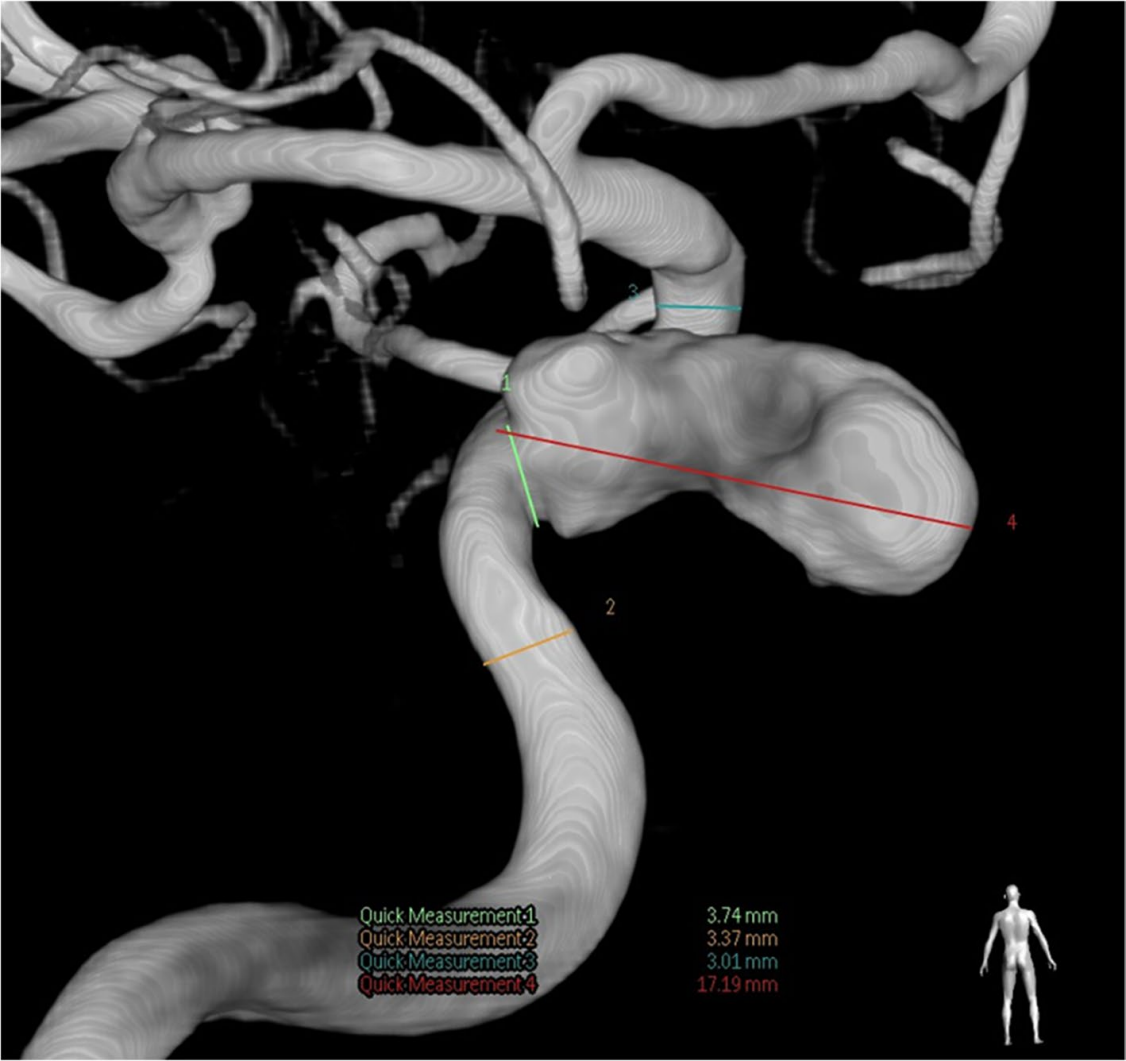
The posterior image of cerebral angiography demonstrates a large (17.19 mm × 8.33 mm × 7.82 mm) aneurysm originating from the cavernous segment of the left ICA

**Fig. 3:**
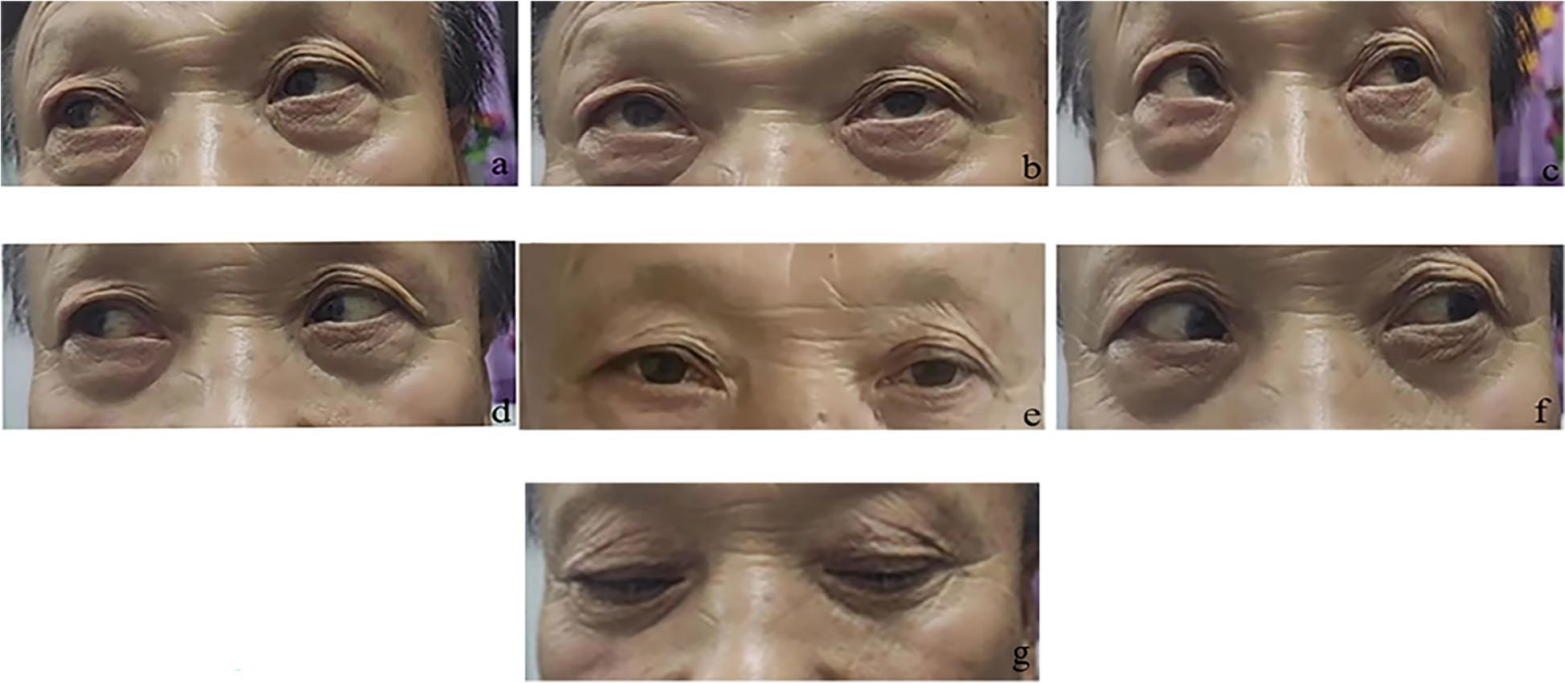
Four months after surgery, neuro-ophthalmologic examination was completely normal. **a**: upper right gaze, **b**: up gaze, **c**: upper left gaze, **d**: right gaze, **e**: primary position, **f**: left gaze, **g**: down gaze

A 62-year-old male farmer was admitted to our hospital with a 2-month history of persistent headache and a 1-week history of ptosis. When the headache started, the patient received analgesic treatment, and the symptoms were relieved to some extent. One week prior to admission, the patient's headache worsened, and was accompanied by ptosis. Brain MRI at a local hospital indicated abnormal signals in the bilateral cavernous sinuses and adjacent meninges. Lumbar puncture showed normal opening pressure and slight leukocytosis (20×10^6^/L reference range 0-8×10^6^/L). Despite hormonal and symptomatic treatment, the patient's symptoms were not relieved. The patient was referred to our hospital for treatment. A previous medical history revealed diabetes for 2 months, with insulin prescribed. Physical examination on admission showed that the patient had paralysis of cranial nerves III, IV, and VI, which presented as bilateral ptosis and bilateral total ophthalmoplegia. He had bilateral exophthalmos and dilated pupils (5 mm) with sluggish pupillary reflexes. The bilateral near reflex and corneal reflex disappeared. The findings of other neurological examinations were normal. Laboratory tests revealed elevated C-reactive protein (60.95 mg/L reference range, 0-5 mg/L), erythrocyte sedimentation rate (37 mm/h reference range, 0-15 mm/h), and reduced luteinizing hormone (1.12mIU/mL reference range, 1.7-8.6 mIU/mL), prolactin (2 ng/mL reference range, 4.04-15.2 ng/mL), testosterone T (0.365 ng/mL reference range, 1.93-7.4 ng/mL), and thyrotropin (0.13 μIU/mL reference range, 0.56-5.91 μIU/mL). The serum myocardial enzyme levels; liver function results; renal function results; D-dimer and homocysteine levels; and autoimmunity markers including antinuclear antibody, anti-neutrophil, cytoplasmic, and rheumatoid factor were within the normal range, and the screening results for syphilis, hepatitis B, hepatitis C, and acquired immunodeficiency syndrome were all negative. Contrast-enhanced MRI showed a mass lesion in the left cavernous sinus (Fig.1A). Pituitary MRI revealed pituitary compression and a right shift of the pituitary stalk under compression (Fig.1B). CTA showed a large aneurysm in the cavernous sinus segment of the left internal carotid artery (approximately 14.2 × 11.7 mm) (Fig. 1C) and no thrombus in the aneurysm cavity (Fig.1D). He was then referred to the Department of Interventional Neurology for endovascular coiling. The aneurysm was embolized using multiple detachable coils. Intraoperative cerebral angiography showed a strip aneurysm of the cavernous sinus segment of the left internal carotid artery with an irregular shape and a size of approximately 17.19 mm × 8.33 mm × 7.82 mm. (Fig. 2). One week after surgery, headache improved significantly, but eye movement was still severely limited, manifested by inability to move in all directions. He still has left ptosis. Four months after the operation, the patient's headache and ptosis disappeared, and eye movements have completely returned to normal (Fig. 3). Due to the limited cooperation of the patient, only part of the eye movement examination images are shown.

## Discussion and conclusion

We describe the rare case of bilateral painful ophthalmoplegia possibly due to a unilateral aneurysm. The cavernous (C4) segment of the internal carotid artery is located medially within the cavernous sinus and is surrounded by a number of intracranial nerves, which includes the oculomotor nerve, trochlear nerve, abducent nerve, and ophthalmic and maxillary branches of the trigeminal nerve [7]. Therefore, an aneurysm of the cavernous segment of the internal carotid artery can cause retro-ocular pain, neuro-ophthalmic signs and symptoms which include diplopia, ophthalmoplegia, trigeminal neuropathy and so on. In our case, based on the patient's symptoms, findings of imaging studies, and failure of hormone therapy, it was likely that the patient's bilateral painful ophthalmoplegia was caused by the cavernous segment aneurysm of the left internal carotid artery.

The exact mechanism of painful ophthalmoplegia caused by an ipsilateral intracranial aneurysm is unclear. The following mechanisms may be speculated: (1) direct compression of the aneurysm, (2) pulsating effect of the aneurysm, (3) perianeurysmal edema, and (4) perianeurysmal inflammation, which was caused by the chronic stimulation of the surrounding tissue by the aneurysm. This stimulation could be compression or it could be blood leaking out of the aneurysm wall [8–11]. In this case, the aneurysm was located in the cavernous segment of the left internal carotid artery, but the patient presented with bilateral painful ophthalmoplegia. So far, the mechanism of action is not clear. In addition, the mechanism of hypopituitarism may be due to direct mechanical compression of the pituitary or aneurysm compression of the superior pituitary artery, leading to pituitary ischemia [12]. Pituitary MRI suggested that the pituitary gland was compressed by the aneurysm, and the pituitary stalk shifted to the right, which corroborates theory.

In conclusion, this case suggests that an internal carotid cavernous segment aneurysm can present with bilateral painful ophthalmoplegia. Given the potentially fatal outcome of aneurysmal rupture [13], any patient who presents with bilateral painful ophthalmoplegia should be promptly screened using CTA or MRA.

## Data Availability

There are no associated datasets for this manuscript. All data generated or analyzed during this study are included in this published article. Related queries can be directed to the corresponding author.

## Abbreviations

CTA: Computed tomography angiography
MRA: Magnetic resonance angiography
MRI: Magnetic resonance imaging
